# Variations in Nurse Practitioner full practice authority in the United States: Difference in difference analysis of access and health Performance at a national level

**DOI:** 10.1016/j.hpopen.2026.100167

**Published:** 2026-03-24

**Authors:** Joyce J. Fitzpatrick, Evelyn G. Duffy, Maxwell Mehlman, Mark Votruba, Joshua A. Gerlick, Catherine M. Dower, Summer Davis, Alicia Plemmons, Allison A. Norful

**Affiliations:** a Marian K. Shaughnessy Nurse Leadership Academy, Frances Payne Bolton School of Nursing Case Western Reserve University, 10900 Euclid Ave, Cleveland, OH 44106-7343, USA; b Case Western Reserve University School of Law, 10900 Euclid Ave, Cleveland, OH 44106-7148, USA; c Case Western Reserve University Weatherhead School of Management, 10900 Euclid Ave, Cleveland, OH 44106-3922, USA; d Catherine Dower Consulting, PO Box 8362, Berkeley, Ca 94707, USA; e Cleveland Clinic, 9500 Euclid Ave, Cleveland, OH 44195, USA; f Knee Regulatory Research Center, West Virginia University, 83 Beechurst Ave, Morgantown, WV 26505, USA; g Columbia University School of Nursing, 630 West 168th Street, Mail Code 6, New York, NY 10032, USA

**Keywords:** Workforce policy, Nurse Practitioner, Full practice authority, Patient Access, Primary Healthcare

## Abstract

**Background:**

Authority over healthcare professional regulation in the United States rests with individual states, resulting in varied timelines and approaches to granting nurse practitioners (NPs) full practice authority (FPA). The impact of these policy changes on healthcare utilization and patient outcomes remains unclear.

**Objective:**

To evaluate the effect of NP full practice authority on healthcare access and patient outcomes across U.S. states.

**Methods:**

We applied a staggered difference-in-differences model using data from state legal statutes and the Medical Expenditure Panel Survey (1997–2018). Analyses included all 50 states and the District of Columbia to assess changes in healthcare utilization—specifically, NP-provided primary care visits, physician visits, and emergency department (ED) use—before and after FPA enactment.

**Results:**

States adopting NP full practice authority experienced a modest but significant increase in NP primary care visits without a corresponding decline in physician visits. Additionally, non-urgent ED utilization decreased in states with FPA, suggesting improved access to primary care. Patient-reported activities of daily living improved modestly, indicating potential health benefits associated with expanded NP authority.

**Conclusions:**

Granting full practice authority to nurse practitioners enhances access to care, optimizes healthcare utilization, and may yield modest improvements in patient outcomes. These findings support the role of FPA policies in strengthening the primary care workforce while maintaining quality of care. Future research should explore long-term impacts and broader health system effects as additional states implement or modify FPA legislation.

## Highlights

• Impact of NP full practice authority on access and outcomes remains unclear.
• FPA adoption increases NP-led visits and lowers non-urgent ED use.
• NP full practice authority boosts access, utilization, and patient outcomes.

## 1 Background

The U.S. healthcare system faces persistent challenges related to high costs and limited access to care, particularly in primary care and in rural and underserved regions.[1], [2] Addressing these issues requires expanding healthcare workforce and maximizing efficiency of existing providers. One policy response has been gradual expansion of legal recognition for nurse practitioners (NPs), allowing them to practice to the full extent of their education and training.[3] Despite growing number of healthcare professionals—including physicians (MDs and DOs), physician assistants (PAs), and NPs—many, particularly physicians, opt for specialties outside primary care and prefer practicing in urban and suburban settings over rural and underserved areas.[4] Nonetheless, the United States (U.S.) has an abundant supply of healthcare providers in absolute numbers and in relation to the general population.[5] However, regulatory barriers in many states continue to limit NPs' ability to independently deliver primary care, affecting overall healthcare accessibility.

As authority over healthcare professional regulation rests with individual states, the U.S. has seen considerable variation in the timeline and approach to granting NPs full practice authority—the ability to diagnose, treat, and prescribe medications without physician oversight. Some states enacted changes more than two decades ago, over half have adopted policies in the past 20 years, and others have yet to implement them.[6], [7] While some states transitioned gradually through incremental changes, others implemented full practice authority more abruptly.[6] This variation in state regulations has created a “natural experiment” that allows researchers to assess whether granting NPs full practice authority improves patient care and access to healthcare. Prior studies have found that states with full practice authority report reduced opioid prescriptions, increased routine checkups, and lower emergency department utilization.[7], [8], [9] However, much of this research has been constrained by a focus on specific populations (e.g., Medicaid patients), localized geographic areas, or binary classifications of full practice authority.[8], [10], [11].

Existing research often fails to account for incremental regulatory changes, instead treating states as either having or not having full practice authority based on a single legislative enactment.[12] In reality, many states gradually broadened NP authority over years, adding discrete privileges—such as prescribing additional drug schedules, signing death certificates, or authorizing independent referrals—before ultimately achieving full practice authority.[13], [14] Staggered changes complicate efforts to identify clear-cut shifts in healthcare access and outcomes. Thus, the purpose of this study was to examine the impact of full practice authority on healthcare access, utilization, and patient outcomes in states where there was an abrupt policy change, with no incremental changes in the four years prior or two years after the policy change. This allows us to more specifically measure the effect of FPA, rather than overlapping policy changes. Using a staggered difference-in-difference model and incidence rate ratio (IRR) analysis, this study evaluates changes in outpatient primary care visits, emergency department utilization, and activities of daily living (ADLs) in states that implemented full practice authority as compared to those that did not.

## 2 Methods

We used a multimethod design by triangulating legal, healthcare, and economic data, each with distinct methodologies and sources. Research phases were conducted sequentially, with legal analysis informing subsequent difference-in-differences analyses.

### 2.1 Legal research: Identifying significant policy changes

The legal research aimed to identify U.S. states that experienced a sharp change in NP scope of practice between 1997 and 2018. This twenty-year period would enable the researchers to use the most current and comprehensive quality and outcomes data sets available at time of study that would subsequently be integrated with the legal data. A jurisdiction was considered to have experienced a sharp change in NP practice if it had passed significant legislation affecting NPs between 1997 and 2018 in a year that was bracketed by relative quiet (no other significant legislation passed) in that jurisdiction for periods before (four years) and after (two years) the significant legislation passed. The study team identified these amounts of time before and after significant legislation as being critical to isolating major significant legislation and avoiding the cloudiness that multiple laws being passed close together can have on interpreting impact of policy. For purposes of this research, significant legislation is described as laws that 1) changed the conditions under which NPs were permitted to diagnose, treat, and refer patients; or 2) changed the conditions under which NPs were permitted to prescribe legend drugs or controlled substances. Legal research for this project was conducted between January and August 2021.

Legal statutes and codes current as of 2021 from all 50 states and the District of Columbia were retrieved from jurisdictional websites, Westlaw, Hein Online, and legislative summaries published in *The Nurse Practitioner* journal. Twenty-nine jurisdictions were identified to have experienced significant changes, mostly having moved to full-practice authority for NPs at some point. Using online legal databases, legislative histories of all identified laws were traced to determine when significant changes had occurred. Each law was coded based on degree of physician oversight required for NPs to diagnose, treat, refer, and prescribe, extent of legislative changes (e.g., removal of supervision or expansion of prescriptive authority), and limitations or qualifications associated with NP authority. Each change to each significant law was also coded to label the variables; the degree of oversight or lack thereof for NPs to diagnose, treat, refer, and prescribe; and respective dates. These data were recorded in a spreadsheet developed with a legal research team.

After the collection and coding of legal information was completed, a review of the yearly articles about state legislation affecting advancing nursing practice in *The Nurse Practitioner* was conducted. This journal provides a legislative summary for all NP-related SOP annually and is contributed by informants in each respective jurisdiction, helping to provide contemporary validation and context for existing and new-at-the-time laws as well as subsequent regulations that would implement the laws on a state-by-state basis.

Analysis of the information collected and coded in the internal database yielded an initial set of 14 states that met the criteria of experiencing a sharp change in scope of practice or prescriptive authority in a year between 1997 and 2018 with no significant legislative changes for the prior four years or the subsequent two years. At research team meetings, a closer look at the data raised questions about other legislative, regulatory or policy developments that could confound the impact of the significant legislation in three of the jurisdictions, leaving eleven states as meeting the criteria and potentially serving as treatment jurisdictions for the proposed economic analysis. The team then identified key informants in each of those jurisdictions with whom interviews could be conducted to confirm the research team’s conclusions and provide subjective context for the respective legal and regulatory environments. Ten interviews were completed. Legal histories and findings were confirmed. Team analyses conducted in August 2021 found three of the jurisdictions to have some remaining questions about legal or regulatory developments that might confound the study so they were no longer included. The whole legal research and analysis resulted in confirming seven states as definite treatment jurisdictions for the economic analyses: Connecticut, Delaware, Idaho, Maryland, Minnesota, Nebraska, and Nevada. These seven states exhibited a clear and significant shift toward NP full practice authority without intervening policies that could confound results. Control states were those that did not experience major legislative changes affecting NP authority during the study period. The final treatment and control groups formed the foundation for difference-in-differences analysis.

### 2.2 Assessing impact of policy changes

The analysis leveraged restricted data from the Medical Expenditure Panel Survey (MEPS)^11^, a nationally representative dataset that includes healthcare utilization data, such as office visits and emergency department use, as well as patient-level information on providers, allowing identification of NP-provided care. The dataset follows selected individuals over two years, facilitating before-and-after comparisons of policy changes. Since MEPS data with geographic identifiers is restricted, access was granted under strict protocols by the Agency for Healthcare Research and Quality (AHRQ). Data analysis was conducted in a secure federal facility over three days in 2023 under AHRQ supervision, with no electronic communication permitted.

The study evaluated whether NP full practice authority influenced access to care, utilization, and outcomes. Access was measured by the rate at which patients visited NPs versus physicians for primary care. Utilization was assessed through emergency department visits and their classification as emergencies or non-emergencies. Outcomes were measured using a composite score of patients' ability to perform activities of daily living (ADLs). Due to privacy restrictions, ADL data were aggregated into a binary measure indicating whether a patient faced significant limitations.

To account for geographic and socio-economic factors, AHRQ approved control variables at the county level, including health professional shortage area (HPSA) designation, demographics (age distribution, racial/ethnic composition), and socio-economic indicators (education levels, median income, insurance coverage, unemployment rates). Although granular geographic data like county-level identifiers were not available, these controls allowed adjustment for local factors influencing access and utilization.

### 2.3 Analytical approach

Two key issues in existing research were addressed: lack of precise estimates for population-level effects of full practice authority and bias in difference-in-differences (DD) models, where gradual policy changes obscure treatment effects.[15] To mitigate these limitations, we applied two approaches: First, the epidemiological approach used an IRR analysis to measure relative frequency of emergency department use, distinguishing between emergency and non-emergency visits. This method accounted for variations in populations and non-random treatment effects. Second, we used a staggered difference-in-differences (SDD) model to evaluate NP full practice authority across states that implemented policy changes at different times. Unlike traditional DD models, SDD accounted for multiple treatment periods, allowing more accurate estimation of policy effects and reducing bias from incremental scope-of-practice expansions.

The final analysis focused on three treatment states—Connecticut (first full year of policy change was 2018), Maryland (2015), and Minnesota (2015)—where full practice authority reforms were identified. Control states were those that had never enacted full practice authority and met the population threshold required for MEPS geographic identifiers. Due to AHRQ confidentiality requirements, state-specific results were anonymized, with treatment states presented as “State 1,” “State 2,” and “State 3” in the tables.

## 3 Results

### 3.1 Access to care – Outpatient visits

Despite excluding smaller states due to population size restrictions, 88.69% of total patient observations were within a previously identified state. Within this sample, 57.4% of patients resided in or near large metropolitan areas, while 15.77% lived in high-poverty areas. Table 1 presents results of the staggered difference-in-differences model, analyzing primary care visits with an NP in states that experienced a sharp policy shift toward full practice authority between 1997 and 2018. The staggered difference-in-difference analysis of NP visits for primary care showed a small but statistically significant increase in overall average treatment effect (ATT) in Model 1 (0.0271; SE = 0.0151), though this effect was not statistically significant in Models 2 and 3. State-level effects varied, with State 1 consistently showing a significant increase across models (Model 1: 0.0507; SE = 0.0262; Model 2: 0.0466; SE = 0.0274; Model 3: 0.0378; SE = 0.0300), while State 2 showed no significant change, and State 3 had a larger but statistically insignificant increase in Model 3 (0.0792; SE = 0.0324). The overall ATT for treatment states represents the policy change impact across three states of interest. Although individual states cannot be identified due to data anonymity restrictions, the baseline model indicates a positive treatment effect in each state (randomly assigned as “State 1,” “State 2,” and “State 3”), confirming that all three states experienced this increase. Column 2 repeats the baseline model while incorporating county HPSA status, showing similar direction and magnitude of results. The most restrictive model, Column 3, accounts for local-level variations in HPSA status, population size, rural classification, and socioeconomic demographics. While not statistically significant, treatment states still exhibited an approximate 1% increase in patient visits to NPs for primary care services in the years following the policy change. A similar analysis examining visits with an NP, PA, or CNM for primary care produced nearly identical results. However, these findings are not included in the main text due to extremely small sample sizes for PA and CNM visits, making them less directly comparable (Supplemental Table 4).

**Table 1:** Staggered Difference-in-Difference of Visits with a Nurse Practitioner for Primary Care.

	Model 1	Model 2	Model 3
Overall ATT	0.0271* (0.0151)	0.0254 (0.0156)	0.0103 (0.0178)
State 1	0.0507* (0.0262)	0.0466* (0.0274)	0.0378 (0.0300)
State 2	0.0024 (0.0229)	0.0029 (0.0235)	−0.0116 (0.0281)
State 3	0.0385 (0.0273)	0.0380 (0.0275)	0.0792 (0.0324)
Control Variables	NA	HPSA Status	HPSA Status, Population, Urban Code, Population Demographics
Observations	5168	5146	5146

***Note*:** Population demographics include descriptors of the county that were merged prior to encryption by AHRQ. These variables include percent of the population above 65, percent of the population that self-identifies as white, percent of the population that self-identifies as Hispanic, proportion of single parent households, median household income, percent of the population without insurance, percent of the population who did not complete high school, percent of the population with a bachelor’s degree or higher, unemployment rate, and labor force participation rate.

To assess relative change in visit frequency between physicians and NPs, we examined overall outpatient visits in Table 2 and Supplemental Table 5. However, due to a change in data classification for physician specialties during the study period, we were unable to distinguish between patient visits to primary care physicians versus specialists. To address the broader question of total outpatient visits, we analyzed visits classified as primary care encounters with either an NP or a physician of any specialty. The analysis of the ratio of MD to NP use in outpatient primary care showed no statistically significant overall ATT in any model (Model 1: 0.0133; SE = 0.0322), with small, non-significant changes at the state level. State 2 showed a consistent upward trend across models (Model 3: 0.0557; SE = 0.0612), while State 3 had a consistent negative effect (Model 3: −0.0364; SE = 0.0616), though none reached statistical significance. While statistically insignificant, findings suggest a slight increase in total outpatient primary care provided by NPs relative to physicians in states that enacted full practice authority. A similar, though also statistically insignificant, increase was observed when limiting the analysis solely to NP visits (Supplemental Table 5), suggesting that full practice authority may have marginally improved overall access to care.

**Table 2:** Staggered Difference-in-Difference of Ratio of Use of MDs to NPs in Outpatient Primary Care (n = 3235).

	Model 1	Model 2	Model 3
Overall ATT	0.0133 (0.0322)	0.0132 (0.0328)	0.0153 (0.0388)
State 1	0.0158 (0.0359)	0.0152 (0.0363)	0.0220 (0.0419)
State 2	0.0473 (0.0500)	0.0504 (0.0510)	0.0557 (0.0612)
State 3	−0.0252 (0.0503)	−0.0277 (0.0512)	−0.0364 (0.0616)
Control Variables	NA	HPSA Status	HPSA Status, Population, Urban Code, Population Demographics

***Note*:** Population demographics include descriptors of the county that were merged prior to encryption by AHRQ. These variables include percent of the population above 65, percent of the population that self-identifies as white, percent of the population that self-identifies as Hispanic, proportion of single parent households, median household income, percent of the population without insurance, percent of the population who did not complete high school, percent of the population with a bachelor’s degree or higher, unemployment rate, and labor force participation rate.

### 3.2 Emergency department use

The impact of NP full practice authority on emergency department (ED) access and utilization (Table 3). The MEPS database categorizes ED visits based on diagnostic codes, allowing us to distinguish between actual emergencies and non-emergency visits (e.g., visits for simple diagnostics). Emergency department utilization was assessed using the epidemiological IRR approach. The States with no practice authority changes IRR represents the incidence rate among control states, while the Expanded Practice Authority States IRR reflects the rate among three treatment states. By pooling and weighting these standardized measures, we assessed whether treatment and control states exhibited significantly different behaviors. Table 3 shows that following full practice authority implementation, emergency department visits in treatment states were more frequently used for accidents and injuries classified as actual emergencies, rather than for non-emergency care, compared to control states. Analyzing total ED visits, we found no significant differences between treatment and control states. Emergency department use outcomes showed minimal differences between Expanded Practice Authority States and states with no practice authority changes groups for both diagnostic purposes (Expanded Practice Authority States IRR = 1.0133) and emergency-related accidents or injuries (Expanded Practice Authority States IRR = 0.9938), with standardized incidence ratios closely aligned (1.0433 and 0.9708, respectively). Homogeneity tests for both outcomes were significant (p < 0.001), suggesting notable variation in treatment effects across groups. These findings suggest that NP full practice authority may be associated with a shift in ED utilization, potentially reducing non-essential visits.

**Table 3:** Incidence Rate Ratios (IRR) for Emergency Department Use.

	Emergency Room Use for Diagnostic Purposes	Emergency Room Use for Accident or Injury Categorized as an Emergency
U.S. States with no practice authority changes	1.0511	0.9640
U.S. States with expanded practice authority changes	1.0133	0.9938
Pooled (direct)	1.0427	0.9703
Standardized Incidence	1.0433	0.9708
Homogeneity	0.0000	0.0000

### 3.3 Quality of care – patient outcomes – activities of daily living

Patient outcomes were examined using restricted MEPS composite score for activities of daily living (ADLs), a key indicator of functional health (Table 4). Additional incidence rate ratio analyses for one-year medication history, including flu vaccination, opioid prescriptions, and antibiotic prescriptions, can be found in Supplemental Table 5.

**Table 4:** Incidence Rate Ratios for Activities of Daily Living.

	Reported Activities of Daily Living as “Good” in Last Month	Reported Activities of Daily Living as “Bad” in Last Month
U.S. States with no practice authority changes	0.9739	1.0215
U.S. States with expanded practice authority changes	1.0027	1.0234
Pooled (direct)	0.9799	1.0219
Standardized Incidence	0.9802	1.0219
Homogeneity	0.0000	0.0000

As with emergency department visits, ADL status was analyzed using the IRR approach. This composite score was the only ADL measure consistently available in the restricted MEPS dataset across the entire sample period. Due to changes in survey questions related to ADLs, individual components could not be assessed separately. Table 4 reveals that treatment states exhibited a higher rate of patients reporting their ADLs as “good” within the past month. Additionally, a slight increase was observed in the proportion of patients from treatment states reporting “bad” ADLs within the same timeframe. However, in aggregate, the increase in patients reporting “good” ADLs in treatment states exceeded the increase in those reporting “bad” ADLs. For activities of daily living, reported functioning as “good” or “bad” in the last month showed negligible differences between states with and without expanded practice authority. The expanded practice authority states IRR for “good” ADLs was slightly above 1 (1.0027), while for “bad” ADLs it was 1.0234, indicating minimal practical effect. As in Table 3, homogeneity tests were statistically significant (p < 0.001), indicating heterogeneity in effects across the population. While these differences are modest, they are statistically significant, suggesting that expanded NP practice authority may provide marginal improvements in patients' ability to perform daily activities.

## 4 Discussion

This study employed a well-grounded yet novel analytical approach to examine the impact of full practice authority for NPs on access to primary health care and patient health outcomes in the U.S. We found that states implementing NP FPA experienced a small increase in outpatient primary care visits with NPs, though effects varied by state and were not consistently statistically significant after adjustment. FPA was also associated with modest shifts in healthcare utilization, suggesting slightly greater NP use in outpatient primary care and fewer non-emergency emergency department visits, while patient functional outcomes (ADLs) showed minimal but slightly favorable changes. In terms of access to care, states that implemented NP full practice authority experienced an overall increase in total outpatient primary care. This contrasts with states that did not expand NP practice authority. The increases in primary care visits appear to stem from higher NP utilization rather than a reduction in opportunities for primary care physicians. While some of these increases were modest or statistically insignificant, the absence of any decline in primary care visits suggests that adopting full practice authority does not displace physician-led care but rather supplements and enhances access to primary care services. This finding is consistent with emerging studies demonstrating that the increased NP supply significantly increases patient access to care.[16], [17], [18].

While the overall number of ED visits per capita was slightly lower in states that had adopted full practice authority for NPs, the composition of those visits changed significantly with NP practice authority. All states continued to experience a mix of ED visits for general or diagnostic purposes (which could have been handled in a primary care setting) and emergencies. However, in states with NP full practice authority, a larger proportion of ED visits were classified as emergencies rather than diagnostic visits, compared to states that had not adopted such laws. Although causation cannot be definitively established, these findings suggest that increasing NP-led primary care access may reduce unnecessary ED utilization. Similar to these findings, a targeted study examining the impact of NP supply across New York State, determined that as NP supply increases, ED utilization and hospitalizations decrease.[8] This has significant implications for healthcare efficiency, as reducing non-urgent ED visits can alleviate overcrowding, lower healthcare costs, and improve emergency care access for those in critical need.[19] Further research should explore whether NP-led primary care directly influences patient decision-making regarding ED use.

Finally, our analysis of ADL measures provides valuable insight into patient health status and functional outcomes. In states that implemented full practice authority, a larger proportion of patients reported improvements in their ability to perform ADLs compared to those reporting declines. Although these gains were modest, they suggest that increasing access to primary and routine care through NP practice authority expansion may yield marginal but meaningful improvements in patient well-being. Ensuring that individuals can manage daily activities independently is a crucial component of overall health and quality of life. Researchers and policymakers have noted it is crucial to develop a well-trained and prepared workforce, strengthen public health initiatives, and address existing disparities and inequities in healthcare.[20] By proactively focusing on alternatives to and expansion of existing regulatory policy frameworks, such as NP SOP, we may better support a healthcare system that fosters improved health outcomes and equitable, goal-oriented care that aligns with the preferences and needs of older adults.

Countries facing aging populations, growing chronic disease burdens, and provider shortages are increasingly turning to expanded NP roles combined with supportive policy frameworks—insights reinforced by new U.S. findings on full practice authority (FPA). In the United Kingdom, Advanced Clinical Practitioners (many of whom are nurses) have been incorporated across general practices to alleviate workforce pressures. A regional qualitative evaluation reported “a high degree of acceptance of the ACP role and affirmation of the important contribution of ACPs to patient care” in the face of rising service demand, while calling for standardized education, governance, and career pathways to solidify effectiveness.[21], [22] In Canada, Nurse Practitioner–Led Clinics (NPLCs) have emerged as a promising model for chronic disease management. In one study, researchers found that the quality of care for patients with diabetes and multimorbidity in NPLCs was high and largely guideline-concordant.[23] Complementary qualitative research from Ontario found that NPLCs enhance access, offer continuity through interprofessional teams, and empower self-management in community-dwelling adults with chronic illness.[24] These structures appear especially well-suited to populations “falling between the cracks” of traditional care models.

The new U.S. evidence in this present paper further strengthens the global case for NP autonomy: using robust staggered difference-in-differences methods over two decades of national data, researchers demonstrated that FPA increases NP primary care utilization without diminishing physician visits, redirects non-urgent emergency department use toward appropriate primary care, and yields modest improvements in patients’ functional health (measured via activities of daily living). While there is debate whether primary care utilization (overuse of services) actually yields better care outcomes, there is evidence that more utilization of care services improves continuity of care, a known determinant of lower mortality.[25], [26] Similarly, higher primary care frequency has been associated with non-urgent use ED use.[27], [28] This can be interpreted that care utilization is most meaningful when paired with measures of continuity, prevention, and patient centered outcomes. Taken together, these findings suggest that NP empowerment, in the form of FPA or analogous regulatory reforms, can supplement existing care capacity, enhance efficiency, and support functional well–being, particularly in settings with aging demographics, chronic disease prevalence, and gaps in provider coverage. This combined evidence offers a compelling policy guide: nations may look to adapt NP role expansion through structured regulatory reform, education, and support systems to achieve measurable health benefits and operational resilience.

Several limitations of this research should be noted. Geographic restrictions limited the analysis to states exceeding the MEPS population threshold. Data anonymization prevented the identification of unique state-specific effects. Additionally, while incremental policy changes in non-treatment states were accounted for, some residual bias may remain. The static measure of the ADL measure (versus assessing change in ADL concurrent with the changes in practice authority) means that the ADL analyses may tell us more about the population served than the effect of the policy changes in practice authority. Despite these constraints, MEPS remains the most comprehensive dataset for evaluating NP policy changes, providing robust insights into the impact of full practice authority on healthcare access and patient outcomes.

## 5 Conclusion

By identifying a select group of states that experienced a sharp policy shift toward NP full practice authority and comparing them to similar states without such changes, this study conducted analyses without confounding effects of incremental legal changes. The findings extend previous evidence by demonstrating that full practice authority for NPs enhances access to care, increases healthcare utilization, and may lead to modest but positive improvements in patient outcomes. Future research should expand the number of treatment states analyzed, potentially leveraging alternative methods to accommodate constraints imposed by federal data limitations. A broader scope could yield more statistically significant findings, further clarifying the long-term impacts of NP full practice authority on healthcare access, utilization, and patient well-being.

## Authors contributions

JF: conceptualization, design, implementation, interpretation, writing and review of final manuscript; EV: conceptualization, design, implementation, writing and review of final manuscript; MM: conceptualization, design, implementation, writing and review of final manuscript; MV: design and analysis plan; writing and review of final manuscript; JG: data analysis, writing and review of final manuscript; CD: conceptualization, design, implementation, writing, review of final manuscript; SD: interpretation, writing and review of final manuscript; AP: data collection, data analysis, writing and review of final manuscript; AAN: interpretation, writing and review of final manuscript.

## Funding Statement

This study was funded by the Charles Koch Foundation and Diana Davis Spencer Foundation.

## CRediT authorship contribution statement

**Joyce J. Fitzpatrick:** Writing – review & editing, Writing – original draft, Project administration, Methodology, Investigation, Funding acquisition, Conceptualization. **Evelyn G. Duffy:** Writing – review & editing, Writing – original draft, Methodology, Investigation, Conceptualization. **Maxwell Mehlman:** Writing – review & editing, Writing – original draft, Resources, Methodology, Investigation, Conceptualization. **Mark Votruba:** Writing – review & editing, Methodology, Investigation, Conceptualization. **Joshua A. Gerlick:** Writing – review & editing, Methodology, Investigation, Formal analysis, Data curation, Conceptualization. **Catherine M. Dower:** Writing – review & editing, Methodology, Investigation, Conceptualization. **Summer Davis:** Writing – review & editing, Methodology, Investigation, Conceptualization. **Alicia Plemmons:** Writing – review & editing, Writing – original draft, Visualization, Methodology, Investigation, Formal analysis, Data curation. **Allison A. Norful:** Writing – review & editing, Writing – original draft, Project administration, Methodology, Investigation.

## Declaration of competing interest

The authors declare the following financial interests/personal relationships which may be considered as potential competing interests: Catherine Dower and Allison Norful were paid consultants on this project. Alicia Plemmons’ institution, Knee Regulatory Research Center received a sub-award grant from Case Western Reserve University to participate in this research.
