# Supplementary material for: Variations in Nurse Practitioner full practice authority in the United States: Difference in difference analysis of access and health Performance at a national level
Source: Health Policy Open. 2026 Mar 24;10:100167. doi: 10.1016/j.hpopen.2026.100167 (PMC13054040; doi:10.1016/j.hpopen.2026.100167)
Supplement: Supplementary Data 1 [file mmc1.docx]

# ***Supplemental Material***

**Supplemental Table 1.** Codes for conditions under which Nurse Practitioners were permitted to diagnose, treat, and refer patients

| **Code** | **Conditions** |
| --- | --- |
| 1 | Physician involvement/oversight always required |
| 2 | Physician involvement/oversight required for some period of time (i.e. “transition period”) |
| 3 | Physician or experienced NP involvement/oversight required for some period of time |
| 4 | Allowed without any oversight or “transition period” requirement |

**Supplemental Table 2.** Codes for conditions under which NPs had prescriptive authority

| **Code** | **Conditions** |
| --- | --- |
| 0 | Not allowed |
| 1 | Allowed but physician involvement/oversight always required |
| 2 | Allowed but physician involvement/oversight required for some period of time (i.e. “transition period”) |
| 3 | Allowed but physician or experienced NP involvement/oversight required for some period of time |
| 4 | Allowed without any oversight or “transition period” requirement |

**Supplemental Table 3.** Staggered Difference-in-Difference of Visits with a Nurse Practitioner, Physician Assistant, or Certified Nurse Midwife for Primary Care

|  | **Model 1** | **Model 2** | **Model 3** |
| --- | --- | --- | --- |
| Overall ATT | 0.0272* (0.0151) | 0.0254 (0.0156) | 0.0103 (0.0178) |
| State 1 | 0.0507* (0.0262) | 0.0466* (0.0274) | 0.0378 (0.0300) |
| State 2 | 0.0024 (0.0229) | 0.0029 (0.0235) | -0.0116 (0.0281) |
| State 3 | 0.0385 (0.0273) | 0.0380 (0.0275) | 0.0792 (0.0324) |
| Control Variables | NA | HPSA Status | HPSA Status, Population, Urban Code, Population Demographics |
| Observations | 5168 | 5146 | 5146 |

**Note:** Population demographics include descriptors of the county that were merged prior to encryption by AHRQ. These variables include percent of the population above 65, percent of the population that self-identifies as white, percent of the population that self-identifies as Hispanic, proportion of single parent households, median household income, percent of the population without insurance, percent of the population who did not complete high school, percent of the population with a bachelor’s degree or higher, unemployment rate, and labor force participation rate.

**Supplemental Table 4.** Staggered Difference-in-Difference of Use of NPs in Outpatient Care (per capita)

|  | **Model 1** | **Model 2** | **Model 3** |
| --- | --- | --- | --- |
| Overall ATT | 0.3763 (0.3168) | 0.4362 (0.3250) | 0.5447 (0.5505) |
| State 1 | 0.3981 (0.4922) | 0.4896 (0.5285) | 0.6854 (0.4808) |
| State 2 | 0.8352* (0.4961) | 0.8532* (0.5010) | 0.8303 (1.0897) |
| State 3 | -0.8600 (0.8296) | -0.7555 (0.8080) | -0.4899 (0.7013) |
| Control Variables | NA | HPSA Status | HPSA Status, Population, Urban Code, Population Demographics |
| Observations | 5566 | 5543 | 5543 |

**Note:** Population demographics include descriptors of the county that were merged prior to encryption by AHRQ. These variables include percent of the population above 65, percent of the population that self-identifies as white, percent of the population that self-identifies as Hispanic, proportion of single parent households, median household income, percent of the population without insurance, percent of the population who did not complete high school, percent of the population with a bachelor’s degree or higher, unemployment rate, and labor force participation rate.

**Supplemental Table 5.** Incidence Rate Ratios (IRR) for 1-Year Medication History

|  | **Received a Flu Vaccine in the Last Year** | **Received an Opioid in the Last Year** | **Received an Antibiotic in the Last Year** |
| --- | --- | --- | --- |
| States with no practice authority changes IRR | 0.9734 | 1.0026 | 0.9999 |
| Treated IRR | 0.9858 | 1.0090 | 0.9879 |
| Pooled (direct) | 0.9758 | 1.0052 | 0.9973 |
| Standardized Incidence | 0.9757 | 1.0039 | 0.9973 |
| Homogeneity | 0.0000 | 0.0000 | 0.0000 |
